# Body composition and depressive/anxiety symptoms in overweight and obese individuals with metabolic syndrome

**DOI:** 10.1186/1758-5996-5-82

**Published:** 2013-12-23

**Authors:** Erika P Guedes, Eduardo Madeira, Thiago T Mafort, Miguel Madeira, Rodrigo O Moreira, Laura MC Mendonça, Amélio F Godoy-Matos, Agnaldo J Lopes, Maria Lucia F Farias

**Affiliations:** 1 Division of Metabology, State Institute of Diabetes and Endocrinology of Rio de Janeiro, Rua Moncorvo Filho 90 - Centro, CEP 20211-340, Rio de Janeiro, RJ, Brazil; 2 Division of Gastroenterology, State University of Rio de Janeiro, Rio de Janeiro, Brazil; 3 Division of Pneumology, State University of Rio de Janeiro, Rio de Janeiro, Brazil; 4 Division of Endocrinology, Federal University of Rio de Janeiro, Rio de Janeiro, Brazil; 5 Division of Rheumatology, Federal University of Rio de Janeiro, Rio de Janeiro, Brazil

**Keywords:** Anxiety, Body composition, Depression, Dual energy X-ray Absorptiometry, Metabolic syndrome, Obesity

## Abstract

**Background:**

Several studies point to a correlation between obesity and the severity of depressive and anxiety symptoms in children and adults, but there are still some controversial points about this association. The aim of this study is to investigate the relationship between body composition and the severity of anxiety/depressive symptoms in overweight and obese individuals with Metabolic Syndrome (MS).

**Methods:**

Fifty patients, 18–50 years old, overweight or obese and with the diagnosis of MS based on the International Diabetes Federation (IDF) criteria were selected for this study. Body composition was evaluated using Dual Energy X-ray Absorptiometry (DXA). Depressive symptoms were evaluated using the Hospital Anxiety and Depression Scale (HADS-Depression) and the Beck Depression Inventory (BDI). Anxiety symptoms were evaluated using HADS-Anxiety.

**Results:**

No correlation was found between depressive symptoms (HADS-Depression or BDI) and Body Mass Index (BMI) (r = 0.01; p = 0.94 and r = −0.12, p = 0.38; respectively), Waist Circumference (WC) (r = −0.06, p = 0.67 and r = −0.22, p = 0.12; respectively), and Waist-to-Hip Ratio (WHR) (r = −0.12, p = 0.40 and r = −0.17, p = 0.23; respectively). Additionally, no correlation was found among anxiety symptoms (HADS-Anxiety) and BMI (r = −0.15, p = 0.27), and WHR (r = −0.17, p = 0.24). In contrast, a significant correlation was found between percentage of total fat (DXA) and HADS-Depression (r = 0.34, p = 0.019) and HADS-Anxiety (r = 0.30, p = 0.039). Additionally, an inverse and strong correlation was found between lean mass (in grams) and HADS-Depression (r = −0.42, p = 0.004), HADS anxiety (r = −0.57, p < 0.0001), and BDI (r = −0.44, p = 0.026).

**Conclusions:**

In individuals with MS, the percentage of body fat, and not central fat, BMI, WC, or WHR, was associated with an increased severity of anxiety and depressive symptoms. In contrast, total lean mass was strongly associated with fewer anxiety/depressive symptoms, suggesting that body composition might be related to psychiatric comorbidity in overweight individuals with MS.

## Background

The prevalence of obesity, depression, and anxiety is growing in the industrialized world [1]. Currently, there is an epidemic of obesity in children and adults resulting mainly from a sedentary lifestyle and overeating. Metabolic Syndrome (MS) may be considered a complication of obesity. In some individuals, the accumulation of adipose tissue in the abdominal cavity (known as visceral obesity) leads to the development of impaired glucose metabolism, hypertension, and dyslipidemia see ref. [2] for review. The combination of these comorbidities increases the risk of complications, including psychopathological conditions [3-5].

Depression and anxiety are two of the most common psychiatric disorders. Several studies point to a correlation between obesity and the severity of depressive and anxiety symptoms in children and adults see ref. [3-5] for review, but there are still some controversial points about this association. While some studies suggest that increased weight *per se* is a major determinant of psychiatric symptoms [5,6], others suggest that fat distribution may be a stronger determinant of this relationship [7,8]. In contrast, it is also plausible to speculate that psychiatric disturbances may interfere with body fat distribution, especially by favoring visceral fat deposits. To the best of our knowledge, most of the studies have used only anthropometric variables in their analysis [5-8]. Dual energy X-ray absorptiometry (DXA) is based on the relative attenuation of two different energy X-rays and produces a three-component model of body composition comprising fat, bone mineral and lean tissue. Scans are relatively quick, involve minimal radiation exposure and also allows for regional analysis and body fat distribution [9]. We are aware of only one previous publication utilizing DXA to evaluate body composition, to test its relationship with the severity of psychiatric symptoms in individuals with MS, and this is the focus of the present study [10]. We therefore hypothesize that body fat and lean mass evaluated separately by DXA could better reflect the influence the association between obesity and depression/anxiety symptoms than anthropometric methods.

### Participants

We evaluated a consecutive sample of fifty patients willing to lose weight who seek treatment for obesity and MS in our Institution and fulfilled eligibility criteria to participate in a study with intragastric balloon (registered at ClinicalTrials.gov; identifier: NCT01598233). The protocol was approved by the Ethics Committee of the State Institute of Diabetes and Endocrinology of Rio de Janeiro, where the patients were recruited, and written informed consent was obtained from each participant. Including criteria: age between 18 to 50 years old, be overweight or obese (Body Mass Index [BMI] ≥ 25 kg/m^2^) and have Metabolic Syndrome (MS) based on International Diabetes Federation (IDF) criteria [11].

Exclusion criteria were as follows: diabetes mellitus type 1 or 2, pregnancy or desire to become pregnant in the next six months, alcoholism, advanced liver disease, end stage renal disease, current or prior Coronary Artery Disease (defined as prior Myocardial Infarction, Stable or Unstable Angina, or coronary revascularization), current or prior cerebrovascular disease (defined as prior Ischemic Stroke, Transitory Ischemic Attack or carotid revascularization), history of psychiatric disorder, current use of antidepressants or other psychiatric medication, use of anti-obesity medications and weight loss treatment in the last 6 months. Patients were examined by an endocrinologist and provided a detailed medical history at baseline evaluation.

### Anthropometrical measures

The following anthropometrical data were registered: body weight (kg), height (m), waist circumference (WC) and waist/hip ratio (WHR). BMI was calculated as weight divided by the square of height (kg/m^2^). Waist circumference (cm) was determined at the midpoint between the lowest rib and the iliac crest. WHR was defined as the ratio of waist girth to the largest circumference of the hips, measured at the trochanter major.

### Evaluation of body composition

Body composition was evaluated by dual energy X-ray absorptiometry [DXA] using a Prodigy-GE densitometer (GE Healthcare, Inc., Madison, WI, USA), and included measurement of body fat content (%), fat distribution and lean mass (grams). The truncal pattern of fat distribution was characterized by preferential fat deposition in the trunk, the android pattern by fat deposition in the abdomen, and the gynoid type by fat deposition in the gluteal–femoral area [12]. Fat Mass Ratio (FMR) was the ratio of the percentage of the trunk fat mass to the percentage of the lower limb fat mass [13].

### Anxiety and depressive symptoms

Anxiety and depression symptoms were assessed by the Hospitalar Anxiety and Depression Scale (HADS), a self-report measurement to assess anxiety and depressive symptoms during the previous week. The items exclude somatic symptoms, avoiding overlap between somatic illness and mood disorders. It includes seven statements on each disorder (HADS-Anxiety and HADS-Depression), and each response consists of a four-point rating scale; a higher score indicates a worse condition [14]. The Beck Depression Inventory (BDI) was also used to measure severity of depression. The instrument comprises 21 questions, each one with four answer options. The total score is the sum of the scores obtained for each individual item [15].

### Statistical analysis

The statistical analysis was performed with GraphPad InStat 3.00 for Windows 95 (GraphPad Software, San Diego, CA, USA). Pearson’s correlation coefficient or Spearman’s correlation coefficient was used to determine the correlations between the anthropometric indicators of weight excess, including body composition, and psychiatric symptoms. Multivariate Linear Regression was used to investigate the impact of age and gender in the severity of anxiety and depressive symptoms. The level of statistical significance was set at 5% (p ≤ 0.05).

## Results

Fifty individuals (40 women), aged from 22 to 48 years old (mean 34.6 ± 7.1), with MS were evaluated. BMI ranged from 29.2 to 53.7 kg/m^2^ (mean 40.0 ± 6.3) and WC from 91 to 147.5 cm (mean 115.4 ± 12.8). The mean value for HADS-Anxiety was 8.4 ± 3.9, for HADS-Depression was 7.1 ± 3.3, and for BDI was 15.0 ± 7.7.

Correlation analysis was used to evaluate the relationship among anthropometric variables (i.e. BMI and WC) and body composition (Table 1). As expected, BMI presented a significant correlation with body fat. However, a significant correlation was also found between BMI and lean mass. On the other hand, a significant correlation was found only between WC and android fat. Unexpectedly, very significant correlations were between WC and lean mass.

**Table 1:** Correlation between anthropometric variables and body composition in obese and overweight individuals with metabolic syndrome

	**BMI (kg/m2)**		**WC (cm)**	
	**R**	**P**	**r**	**p**
Total fat (%)	0.52	0.0002	0.18	0.23
Android fat (%)	0.48	0.0008	0.34	0.021
Gynoid fat (%)	0.43	0.0027	0.10	0.48
Trunk fat (%)	0.38	0.0086	0.16	0.28
Total lean mass (g)	0.38	0.0082	0.65	<0.001
Gynoid lean mass (g)	0.30	0.039	0.51	<0.003
Android lean mass (g)	0.44	0.0024	0.65	<0.001

BMI = Body Mass Index; WC = Waist Circumference.

No correlation was found between depressive symptoms (HADS depression) and BMI, WC and WHR. Also, no correlation was found among anxiety symptoms (HADS anxiety) and BMI and WHR. A negative correlation was found between anxiety symptoms and WC. Very similar results were also yielded with BDI. No correlation was found between BDI and BMI, WC and WHR. In contrast, several parameters of body composition measured by DXA were significantly associated with psychiatric symptoms, especially anxiety. These data are shown in Table 2.

**Table 2:** Correlation between body composition and anxiety/depressive symptoms in overweight and obese individuals with metabolic syndrome

**Parameters**	**HADS-Anxiety**		**HADS-Depression**		**BDI**	
	**r**	***p***	**r**	***p***	**r**	***p***
BMI (kg/m^2^)	−0.15	0.27	0.01	0.94	−0.12	0.38
Waist circumference (cm)	−0.29	0.038	−0.06	0.67	−0.22	0.12
Waist-to-hip ratio	−0.17	0.24	−0.12	0.40	−0.17	0.23
Total fat (%)	0.30	0.039	0.34	0.019	0.22	0.13
Android fat (%)	0.15	0.32	0.25	0.087	0.18	0.22
Gynoid fat (%)	0.26	0.083	0.32	0.029	0.21	0.16
Trunk fat (%)	0.26	0.078	0.32	0.031	0.24	0.11
FMR	−0.27	0.068	−0.27	0.065	−0.11	0.45
Total lean mass (g)	−0.57	<0.0001	−0.42	0.004	−0.44	0.027
Gynoid lean mass (g)	−0.50	<0.0001	−0.33	0.024	−0.34	0.22
Android lean mass (g)	−0.47	<0.0001	−0.37	0.010	−0.44	0.026

BMI = Body Mass Index; g = grams; FMR = Fat Mass ratio; HADS = Hospital Anxiety Depression Scale; BDI = Beck Depression Inventory.

Linear regression was used to investigate whether age and gender would interfere with the association among Total Body fat, Total Lean Mass and anxiety/depressive symptoms. The inverse correlation between Total Lean Mass and HADS-Anxiety remained significant even after adjustment for age and gender (*p* = 0.019). Similar results were yielded with Total Lean Mass and HADS-Depression, although with borderline significance (*p* = 0.06).

## Discussion

The relationship between obesity and mood disorders (especially depression) has been extensively studied in recent years, and a bidirectional association seems unequivocal [4]. However, most of the studies have only evaluated indirect measurements of adiposity, such as BMI and WC [5-8]. These anthropometric variables, although largely used in clinical practice, may neither indicate the total body fat nor differentiate lean from fat mass. In the present study, we used DXA to determine total lean mass and the percentage of body fat and correlated these parameters with depressive and anxiety symptoms in overweight individuals with MS. Our most relevant results were the following: i – a significant correlation was found between BMI and both lean and fat mass; ii – overall, no correlation was found among anthropometric variables and psychiatric symptoms; iii - there was a direct correlation between the percentage of body fat and the severity of depressive and anxiety symptoms; and iii – there was a strong and inverse correlation between lean mass and specific psychiatric symptoms.

The relationship between total fat and psychiatric symptoms in overweight individuals is of great relevance for clinical practice. Recently, a few studies have demonstrated that depressive symptoms may be an important predictor of abdominal obesity [10,16]. The most acceptable hypothesis for this relationship involves the hypothalamus-pituitary-adrenal (HPA) axis. As a chronic stressor, mood disorders may change cortisol secretion by stimulation of the HPA axis. This elevation in serum cortisol can increase abdominal fat deposition, promoting glucose intolerance and hypertension [16,17]. However, the lack of correlation between depression and visceral fat has been demonstrated in the elderly [18]. In contrast, we could demonstrate that the total amount of body fat may be more related to psychiatric symptoms than central fat *per se*. Because only patients with MS were included in the present study, we may speculate that we have already included patients with increased visceral fat.

One unexpected finding was the inverse relationship found between anxiety symptoms and WC. We could not find a reasonable explanation for this novel finding and we may not exclude that this may be only a false positive result. Further studies are necessary to replicate and clarify this issue.

The existence of a strong and inverse correlation between lean mass and anxiety/depressive symptoms in overweight individuals with MS, even more significant than the correlation between body fat and these same symptoms, is a novel finding with major implications. Body Mass Index is the anthropometric variable frequently used in clinical research to investigate the relationship between weight excess and psychopathology. However, major limitations of BMI include that it cannot differentiate lean from fat mass excess [19] and also that there may exist important ethnic-specific differences with this parameter [20]. These limitations may be of particular relevance in men, who may present increased BMI related to muscle hypertrophy and not fat. Therefore, the use of BMI may not reflect a simple increase in total fat, and this finding may be an important confounding bias in several studies that fail to find correlations between BMI and psychiatric disorders [21,22].

Unfortunately, our study was not powered to determine a causal relationship between lean mass and anxiety/depressive symptoms in overweight individuals with MS. We may, however, raise the hypothesis that an increase in lean mass may indicate a healthier individual and therefore be a protective factor for depressive and anxiety symptoms. This hypothesis has already been partially supported by findings of Wagner et al. [23] and Wallymahmed et al. [24] in different populations, and recently by Gubata et al. [21] in a young population. In contrast, one may also speculate that patients with a better psychological profile (i.e., lower rates of psychiatric symptoms) may be more prone to physical activity, which would lead to a significant increase in muscle mass. Lastly, an inverse situation also seems plausible. It has already been demonstrated that older patients with reduced lean mass (sarcopenia) may present an increased prevalence of depressive symptoms [25]. Similar results were obtained by Kress et al. [26], who have demonstrated that underweight men in the U.S. military active service had increased odds of depressive symptoms. It seems reasonable to speculate that in this population, underweight men would indicate men with diminished lean mass.

Our study has a few limitations. First of all, only a small number of overweight and obese patients with MS were evaluated. This is a very selective population and further studies are necessary to confirm whether our findings would also be applicable for different populations (e.g. lean individuals, obese individuals without MS, morbidly obese patients). Second, as only 10 individuals were male, we could not determine the impact of gender in the relationship between body composition and psychiatric symptoms. Third, Prodigy densitometer does not distinct between subcutaneous and visceral fat, which might have allowed for a more thorough analysis of the role of each type of fat in the present study. Finally, physical activity might be an important confounding variable in the relationship between body composition and psychiatric symptoms. Although we did not include a specific instrument to measure physical activity, this information was obtained in the initial evaluation. None of the participants was practicing regular physical activity in the 6 months prior to study enrollment. Therefore, we may speculate that physical activity may not be an important determinant of our findings.

## Conclusion

In conclusion, in overweight and obese individuals with MS, psychological status were directly correlated with total body and regional fat, and inversely related to lean mass. Taken together, these results suggest that the relationship between psychiatric symptoms and body weight should be carefully interpreted when only anthropometric variables are considered, especially if these variables are unable to differentiate lean from fat mass. Further and prospective studies are necessary to clarify the impact that increased lean mass, especially if associated with physical activity, would have in the psychopathological profile of larger populations.

## Competing interests

The authors have no competing interests to report.

## Authors’ contributions

EPG: Conception and design of the study, acquisition of data, drafting of the manuscript. EM: Conception and design of the study, acquisition of data, drafting of the manuscript. TTM: Conception and design of the study, acquisition of data, drafting of the manuscript. MM: Conception and design of the study, carried out DXA exams and interpretation of findings, drafting of the manuscript. ROM: Conception and design of the study, statistical analysis, interpreatation of data, drafting of the manuscript. LMCM: Carried out DXA exams and interpretation of findings, drafting of the manuscript. AFGM: supervised the entire project and provided intellectual feedback throughout. AJL: Conception and design of the study, interpretation of data, drafting of the manuscript. MLFF: supervised the entire project and provided intellectual feedback throughout. All authors read and approved the final manuscript.
